# Editorial: Methods in predictive toxicology 2023

**DOI:** 10.3389/fphar.2025.1556352

**Published:** 2025-02-25

**Authors:** Sankalp Jain, Serena Manganelli, Vitalina Gryshkova, Maria Armanda Rodrigues, Aniket Magarkar

**Affiliations:** ^1^ National Center for Advancing Translational Sciences (NCATS), National Institutes of Health, Rockville, MD, United States; ^2^ Independent Researcher, Lausanne, Switzerland; ^3^ Non-Clinical Safety Evaluation, UCB, Brussels, Belgium; ^4^ Global Health and Tropical Medicine, GHTM, Associate Laboratory in Translation and Innovation Towards Global Health, LA-REAL, Instituto de Higiene e Medicina Tropical, IHMT, Universidade NOVA de Lisboa, UNL, Lisbon, Portugal; ^5^ Boehringer Ingelheim Pharma GmbH & Co. KG, Biberach an der Riß, Germany

**Keywords:** predictive toxicology with AI, pharmacovigilance, machine learning, data-driven decision making, drug development, drug safety, computational modeling

## Introduction

Predictive toxicology is a pivotal field that bridges the gap between experimental findings and risk assessment, enabling the anticipation and mitigation of adverse drug reactions, and selection of safer drug candidates early in drug discovery and development (Amorim et al., 2024; Tonoyan and Siraki, 2024). As the complexity of chemical substances and pharmaceuticals continues to grow, so does the need for innovative methods to predict their potential toxicities (Pognan et al., 2023). Moreover, modulating novel proteins with limited knowledge of safety profiles demands a robust integration of computational and experimental approaches to gain mechanistic insights into potential adverse outcomes.

This Research Topic, part of the Methods in Pharmacology series, highlights significant advancements in computational modeling, mechanistic insights, and experimental methodologies that enhance predictive toxicology. The articles in this Research Topic demonstrate how these tools, combined with omics technologies and pharmacovigilance data, refine toxicity predictions, reduce reliance on animal testing, and improve our understanding of toxicological mechanisms. Together, these approaches aim to ensure safer therapeutic interventions for patients.

## Mechanistic insights through omics and computational modeling

Advancements in omics technologies and computational methods offer deeper mechanistic insights into toxicological processes. Two studies in this Research Topic leverage these approaches to unravel the underlying mechanisms of drug-induced toxicity.

The study “Potential mechanisms underlying podophyllotoxin-induced cardiotoxicity in male rats: toxicological evidence chain (TEC) concept” Ma et al. employs an integrative framework combining transcriptomics and targeted metabolomics to explore the cardiotoxic effects of podophyllotoxin (PPT). By constructing a toxicological evidence chain (TEC), the researchers identify key pathways involved in PPT-induced cardiac injury, including oxidative stress, apoptosis, inflammatory responses, and disruptions in energy metabolism. This comprehensive approach not only elucidates the molecular mechanisms of PPT cardiotoxicity but also provides a template for investigating other compounds with complex toxicological profiles.

In “Quantum chemical calculations of nitrosamine activation and deactivation pathways for carcinogenicity risk assessment,” Göller et al. the authors use quantum chemical calculation to assess the carcinogenic potential of N-nitrosamines, a class of compounds of significant concern due to their presence as impurities in pharmaceuticals. By calculating and comparing activation energy profiles of carcinogenic and non-carcinogenic nitrosamines, they uncover distinct differences in their reactivity and stability. This computational approach predicts mutagenic and carcinogenic risks associated with N-nitrosamines, demonstrating the power of *in silico* methods in regulatory decision-making.

## Utilizing pharmacovigilance tools to detect emerging drug safety signals

Pharmacovigilance plays a crucial role in detecting and analyzing adverse drug reactions post-marketing. Two studies in this Research Topic exemplify how large-scale data analysis can provide invaluable insights into drug safety.

In “Osteonecrosis of the jaw in patients with clear cell renal cell carcinoma treated with targeted agents: a case series and large-scale pharmacovigilance analysis,” Wang et al. the authors investigate the association between tyrosine kinase inhibitors (TKIs), immune checkpoint inhibitors (ICIs), and osteonecrosis of the jaw (ONJ). Utilizing the FDA Adverse Event Reporting System (FAERS), they perform disproportionality analyses to detect signals indicating a higher risk of ONJ with specific agents like sunitinib and lenvatinib. This study underscores the importance of continuous monitoring and data mining in identifying rare but serious adverse events, contributing to safer therapeutic strategies for patients with renal cell carcinoma.

Similarly, the article “Post-marketing safety evaluation of lurbinectedin: a pharmacovigilance analysis based on the FAERS database” Li et al. focuses on lurbinectedin, a novel agent approved for metastatic small-cell lung cancer. The researchers employ multiple algorithms to assess adverse event reports and identify known toxicities such as hematological effects and gastrointestinal symptoms. Notably, they also detect a new safety signal—tumor lysis syndrome—highlighting the dynamic nature of drug safety profiles post-approval. This work demonstrates how pharmacovigilance data can reveal emerging risks and guide clinicians in monitoring and managing side effects.

## Future directions and implications

The studies featured in this Research Topic highlight the evolving landscape of predictive toxicology, where interdisciplinary approaches are essential. The integration of pharmacovigilance data with mechanistic studies provides more precise toxicity predictions and contributes to the development of safer drugs. Looking ahead, future research should expand on these methods, by incorporating artificial intelligence and machine learning to analyze complex datasets and identify subtle patterns. Efforts to standardize protocols and promote data sharing will accelerate progress and foster interdisciplinary collaboration. Additionally, the adoption and advancement in high-throughput omics technologies and computational modeling promise to reduce animal testing by providing alternative approaches to assess toxicities. By understanding the molecular basis of toxic effects, researchers can design targeted interventions and develop predictive models that are both accurate and tailored to specific mechanisms, drug classes, or target contexts.

## Conclusion

This Research Topic exemplifies how innovative methods are transforming predictive toxicology. By leveraging pharmacovigilance large-scale data analysis with mechanistic insights, these studies contribute significantly to uncovering toxicological insights and drug safety. We thank all the authors for their valuable contributions and the reviewers for their critical feedback. We hope that this Research Topic will inspire further research and dialogue, ultimately advancing the field of predictive toxicology and enhancing its impact on public health.
